# Evidence of high-altitude adaptation in the glyptosternoid fish, *Creteuchiloglanis macropterus* from the Nujiang River obtained through transcriptome analysis

**DOI:** 10.1186/s12862-017-1074-0

**Published:** 2017-11-23

**Authors:** Jingliang Kang, Xiuhui Ma, Shunping He

**Affiliations:** 1 The Key Laboratory of Aquatic Biodiversity and Conservation of Chinese Academy of Science, Institute of Hydrobiology, Chinese Academy of Sciences, Wuhan, Hubei 430072 China; 2 University of the Chinese Academy of Science, Beijing, China; 3 College of Animal Science, Guizhou University, Guiyang, Guizhou 550025 China

**Keywords:** *Creteuchiloglanis macropterus*, The Nujiang River, Transcriptome, High-altitude adaptation, Gene expression

## Abstract

**Background:**

Organisms living at high altitudes face low oxygen and temperature conditions; thus, the genetic mechanisms underlying the adaptations in these organisms merit investigation. The glyptosternoid fish, *Creteuchiloglanis macropterus* mainly inhabits regions with gradual increases in altitudes along the Nujiang River and might serve as an appropriate evolutionary model for detecting adaptation processes in environments with altitude changes.

**Results:**

We constructed eleven RNA-sequencing (RNA-seq) libraries of *C. macropterus* collected from five locations at different altitudes to identify the genetic signatures of high-altitude adaptation. The comparative genomic analysis indicated that *C. macropterus* has an accelerated evolutionary rate compared with that of fishes in the lowland, and fishes at higher altitudes might evolve faster. Functional enrichment analysis of the fast-evolving and positively selected genes, differentially expressed genes and highly expressed genes, showed that these genes were involved in many functions related to energy metabolism and hypoxia.

**Conclusions:**

Our study provides evidence of high-altitude adaptation in *C. macropterus*, and the detected adaptive genes might be a resource for future investigations of adaptations to high-altitude environments in other fishes.

**Electronic supplementary material:**

The online version of this article (10.1186/s12862-017-1074-0) contains supplementary material, which is available to authorized users.

## Background

Hostile environments, such as high altitudes, cold climates, low concentrations of oxygen and harsh ultraviolet radiation, pose a challenge to organism survival [1–3]. However, certain animals living on the Qinghai-Tibetan Plateau (QTP), such as humans, yaks and Tibetan antelopes, have adapted to these severe conditions. Exploring these organisms’ adaptations to high altitudes has received considerable attention in the field of evolutionary biology, and many genomic characteristics have been detected in mammals (e.g., wolves [4], Tibetan mastiffs [5] and Tibetan pigs [6]).

Due to the rapid development of sequencing techniques, particularly high-throughput sequencing, the detection of genetic adaptive characteristics has become increasingly accessible. However, compared with the surging number of genome sequencing studies on endothermic terrestrial vertebrates, studies exploring adaptations in fishes that inhabit high-altitude environments are scarce. As the three main high-altitude fish lineages on the QTP, glyptosternoid, schizothoracinae and triplophysa are considered excellent models with which to study high-altitude environments in fishes. Based on a comparison with other fish species that inhabit lowland areas and a transcriptome analysis, the genetic adaptation characteristics were successively identified in a schizothoracinae species [7], three triplophysa fish species [8] and three glyptosternoid fish species [9]. However, all previous studies of fishes only analysed the evolutionary differentiation between fish species inhabiting high versus low altitudes, while the evolutionary fluctuations along a gradient of increasing altitudes are still not clear. Previous studies examined only one sample of each species, which might induce occasional errors and omit important information related to high-altitude adaptations. In addition, the previous studies only focused on calculating and comparing the evolutionary rates of genes, and they ignored gene expression, which is a direct response of the organisms to the environment. Therefore, a more comprehensive analysis is still necessary to identify the genetic mechanisms underlying fish adaptations to high-altitude environments.

*C. macropterus*, a glyptosternoid fish, has large and flat pectoral fins and tends to attach to rocks in the rapidly flowing water. In the present study, *C. macropterus* is mainly distributed in limited regions along the Nujiang River in Yunnan Province in China, which flows above the Southwest Mountain Region at altitudes of 1000–2000 m. Through comparisons with two other catfish species (*Pelteobagrus fulvidraco* from the lowland (< 100 m) and *Glyptosternon maculatum* from higher altitudes (3800–4000 m) [9]), *C. macropterus* could be used to compare the evolutionary rates of genes among species at different altitudes. Therefore, we could utilize the three fish to investigate whether the evolutionary rates would be accelerated with the increased altitude. In consideration of the continuous distribution at different altitudes along the Nujiang river of *C. macropterus*, it might be appropriate to explore fluctuations of gene expression at increased altitudes. By estimating the differentially expressed genes among samples at different altitude, we might detect relevant genomic signals of high-altitude adaptation.

Here, to detect the genomic characters of high-altitude adaptation in *C. macropterus*, we sampled tissues (i.e., brain, heart, liver and spleen) from *C. macropterus* collected in five locations of the Nujiang river in Yunnan province at an altitude from 874 to 1972 m, and we constructed eleven RNA sequencing (RNA-seq) libraries together. First, a relatively intact transcriptome of *C. macropterus* was assembled by integrating all the clean reads. Second, to investigate the evolutionary rates in different altitudes, we detected the orthologues among *C. macropterus*, *P. fulvidraco* and *G. maculatum*, and identified their evolutionary rate respectively, fast-evolving genes (FEGs) and positively selected genes (PSGs). The mutual genes (FPGs) of FEGs and PSGs in the *C. macropterus* lineage would be regarded as putative genes, which might involve with the high-altitude adaptation of *C. macropterus*. Third, to figure out the fluctuations of gene expression along increased altitude in *C. macropterus*, we explored sample clustering and gene expressions, and then identified differentially expressed genes (DEGs) among clusters. And in view of genes that related with high-altitude adaptation might be highly expressed in organisms at highland [10], we extracted the highly expressed genes (HEGs) from tissues that sampled from high altitudes. Finally, to confirm whether FPGs, DEGs and HEGs have correlated with high-altitude adaptation, we performed a gene function enrichment analysis on the three gene lists, and investigated the genes that were enriched in functions associated with high-altitude adaptation, which might be important for *C. macropterus* to survive at high altitude.

## Methods

### Fish sampling and sequencing

**Fig. 1:**
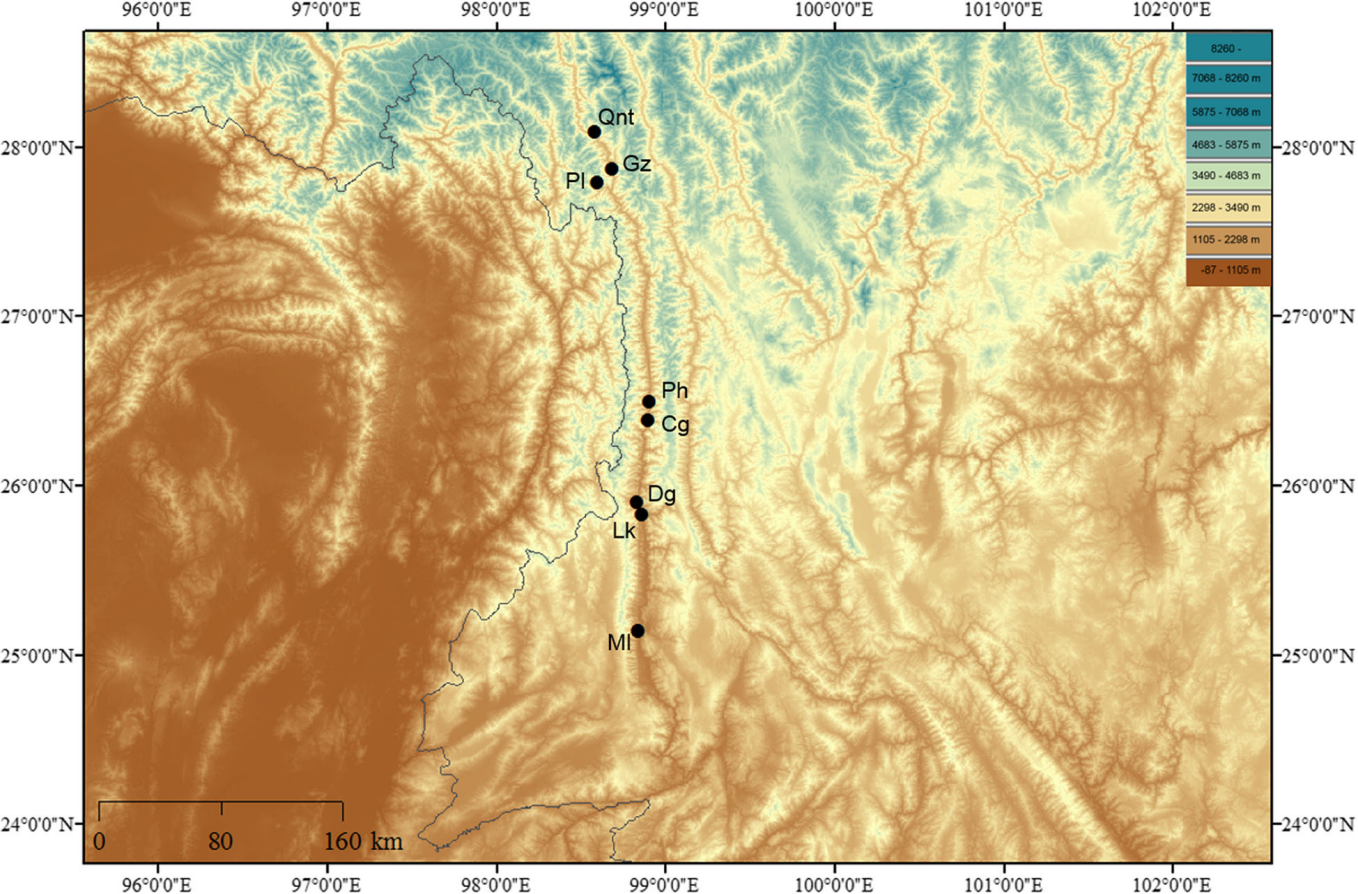
Map showing the locations of the 8 sites where *C. macropterus* samples were involved in this study. Qiunatong (Qnt), Geza (Gz), and Pula (Pl) were located in the up tributaries, Pihe (Ph), Chengan (Cg), Denggeng (Dg) and Liuku (Lk) were located in the mid stem, Mangliu (Ml) was located in the low tributaries. Among these localities, Gz, Pl, Cg, Dg and Ml have sampled for the constructions of RNA-seq libraries. Map was created in ArcGIS version 10.1 and modified in Microsoft Office

All animal experiments were approved by the Ethics Committee of the Institute of Hydrobiology, Chinese Academy of Sciences. We sampled *C. macropterus* from four geographical regions (Yunnan Province, China) in the Nujiang River Basin. The samples were collected from Geza (Gz), Pula (Pl), Chenggan (Cg), Denggeng (Dg) and Mangliu (Ml) (Fig. 1, Table 1). Individuals from Pl and Gz were sampled in upstream tributaries, while individuals from Cg and Dg were sampled in the main stem and an individual from Ml was sampled from a downstream tributary. The heart, liver, brain, kidney and spleen from the *C. macropterus* collected in these locations were sampled and immediately stored in RNA-later (Qiagen, Limburg, the Netherlands) according to the manufacturer’s instructions. In addition, Qiunatong (Qnt), Gz, Pihe (Ph), Liuku (Lk) and Ml (Fig. 1) were the localities that were used to construct 2b–RAD libraries in our previous study.

**Table 1:** Sample collection data of *C. macropterus* in the Nujiang River Basin, including geographic region, coordinates, collection date and *N* (number of individuals sampled per locality), the code is abbreviation for the geographic region

geographic region	code	coordinates	Altitude (m)	Collection date	N
Pula	Pl	27°47′29″N, 98°35′31″E	1972	Oct/2015	1
Geza	Gz	27°52′23″N, 98°40′51″E	1621	Oct/2015	3
Chenggan	Cg	26°23′11″N, 96°53′50″E	937	Oct/2015	1
Denggeng	Dg	25°54′21″N, 98°49′37″E	933	Oct/2015	1
Mangliu	Ml	25° 08′37″N, 98°50′09″E	874	Oct/2015	1

Total RNA was extracted from each tissue of each sample using the TRIzol reagent (Invitrogen). The cDNA libraries were constructed using a TruSeq® RNA sample prep kit (Illumina) according to the manufacturer’s protocol. Finally, we constructed seven mixed libraries (equal quantities of tissues) of samples collected from each location and four non-mixed libraries (heart, liver, brain, kidney and spleen) of samples obtained from Gz. After the quality of the cDNA libraries was assessed, paired-end sequencing was performed using the Illumina HiSeq 2500 platform. All sequence reads were deposited in the National Center for Biotechnology Information (NCBI) Sequence Read Archive database under bioproject number PRJNA380490.

### Transcriptome assembly and annotation

The raw reads were processed to remove reads with sequencing adaptors, unknown nucleotides and low quality (quality scores <20). All subsequent analyses were based on these filtered reads. The transcriptome was assembled using TRINITY [11] with the default parameters. Then, the CD-HIT-EST software [12] was used to remove the redundancy and all the contigs under 600 bp with an identity threshold of 95%.

We first combined all the clean reads together to obtain an integrated transcriptome. After removing the redundancy, the unigenes were annotated by searching against the GenBank non-redundant (nr) database (downloaded from NCBI on 29 April 2016) using the BLASTX algorithm. The putative functions of the assembled unigenes were assigned using BLAST2GO suit [13], and the TransDecoder program (http://transdecoder.github.io/) was applied to obtain the open reading frames (ORFs) of genes.

### Orthology determination and alignment

We used HaMStR v.13.2.6 [14] to construct a core-orthologue database which included eight species including zebrafish (*Danio rerio*), medaka (*Oryzias latipes*), tetraodon (*Tetraodon nigroviridis*), fugu (*Takifugu rubripes*), stickleback (*Gasterosteus aculeatus*), cod (*Gadus morhua*), platyfish (*Xiphophorus maculates*), and tilapia (*Oreochromis niloticus*) [8]. Of the eight species, 16,472 one-to-one orthologues were obtained using Biomart [15] in Ensembl (release 89). The longest transcript was chosen when more than one corresponding orthologue was identified. Zebrafish was used as a representative model to identify the corresponding orthologues in three catfish from different altitudes (*C. macropterus*, *P. fulvidraco* and *G. maculatum*). Each orthologous gene set was aligned using GeneWise [16], and trimmed using Gblocks [16] with the parameter “-t = c”. We further deleted all gaps and “N” from the alignments to reduce the effect of ambiguous bases on the inference of positive selection. After the trimming process, alignments shorter than 150 bp (50 codons) were filtered for the subsequent analyses.

### Phylogenetic tree construction and estimation of the evolutionary rate

By performing a maximum-likelihood (ML) analysis using RAxML 7.0.3 [17], we constructed a phylogenetic tree of the abovementioned four species with the concatenated sequences of the orthologues. In total, 1000 nonparametric bootstrap replicates were performed using the GTRGAMMA substitution model. The best-scoring ML tree in terms of the branch lengths and bootstrap support values was obtained and used in the subsequent analyses.

To estimate the lineage-specific evolutionary rates in each species, the codeml program in the PAML package [18] was run on each orthologue using the free-ratio model (model = 1). Based on a previous study [19], the parameters, including the dN, dS, dN/dS, N*dN, and S*dS values, were obtained for each branch and the genes were discarded if N*dN or S*dS < 1 or dS > 2. Then, we calculated the dN/dS of the concatenated sequence of all orthologs. The mean values of dN/ds were based on each orthologue and ten randomly chosen orthologues [7]. Comparisons of the evolutionary rates in each lineage were conducted using the Wilcoxon rank sum test. The Gene Ontology (GO) terms that involved more than five orthologues were retained to calculate the mean value of Ka/Ks and identify the lineage-specific accelerated GO categories. The Wilcoxon rank sum test was used to identify the GO categories with significantly higher dN/dS values.

### Estimation of genes with accelerated evolutionary rates

The fast-evolving genes (FEGs) and positive selected genes (PSGs) were estimated using the PAML package. The terminal branch of the three catfish species was respectively set as the foreground branch. We used the branch model in codeml to detect the FEGs by comparing the null and alternative models. The null model hypothesized that all tree branches evolved at the same rate (i.e., the same ω) and the alternative model assumed that the foreground branch could evolve at a different rate. A likelihood ratio test (LRT) with df = 1 was used to discriminate between the null and alternative models for each orthologue. We corrected for multiple testing by applying the false discovery rate (FDR < 0.05) method [20] implemented in R. Genes were considered putative FEGs if the ω values of the foreground branch were higher than those of the background branches and their FDR-adjusted *P*-values were less than 0.05.

A positive selection analysis was also performed based on all the orthologues using the branch-site model (model = 2, Nsites = 2) [19] in the codeml program of PAML. A comparison was also conducted between the null and alternative models. The null model assumed that no positive selection occurred on the foreground branch (dN/dS = 1, modelA1, fix_omega = 1 and omega = 1.5), and the alternative model assumed that sites on the foreground branch were under positive selection (dN/dS > 1, modelA2, fix_omega = 0 and omega = 1.5). An LRT was performed to compare the two models and to calculate the statistics (2△ln). After a Chi-square statistical analysis was performed and adjusted according to the FDR, the genes with an adjusted *P*-value less than 0.05 were considered candidates for positive selection.

Genes in the overlap of FEGs and PSGs (FPGs) were considered as adaptive genes for the following functional enrichment analysis.

### Gene expression estimation

The high-quality RNA-seq reads of the samples were individually mapped against the reference using BOWTIE2 [21] with default parameters for the subsequent expression estimation using RSEM [22]. The expression levels were calculated as fragments per kilobase of exon per million reads mapped (FPKM) using log2-transformed and a mean-centred normalized read count. As altitude increasing, some important genes that were related with high altitude adaptation might express more in highland than that in lowland. To characterize the expression patterns, the top 500 highly expressed genes (HEGs) in each sample were utilized to obtain the related expression profiles using the pheatmap package in R [23]. Based on the gene expression profile, the top 400 HEGs in the tissues from high altitudes would be considered as putative adaptive genes. Through the function enrichment analysis of these genes, we might detect some relative genomic signals that be important for the high altitude adaptation of *C. macropterus*.

### Clustering analyses of samples

To estimate the clustering of the samples along the Nujiang River Basin, we performed principal component analysis (PCA) using DESeq2 [24] based on the gene expression of all the samples. In consideration of few samples in the RNA-seq of the present study, we also used 2b–RAD data of the *C. macropterus* populations [15] to identify the population genetic structure, where the assemble transcriptome were used as reference. Therefore, the genetic structure would be estimated through single nucleotide polymorphism (SNP) of mapped tags, which were associated with the expressed genes. An IIB restriction-site-associated DNA (2b–RAD) analysis of the six populations was performed as described in our previous research [25]. All SNP variants were exported into the PLINK format (PLINK v1.0.7) [26] and used to estimate the genetic structure using ADMIXTURE v1.23 [27]. The postulated number of ancestral population K was set to 1–10. In addition, a Discriminant Analysis of Principal Components (DAPC) was applied to provide a visual evaluation of the genetic structure of these populations of *C. macropterus*, using the R package adegenet [28]. The sampling localities were used as prior groups, and all loci that were detected with SNPs were used as inputs [29].

### Differential expression analysis

We searched for pairwise differentially expressed genes (DEGs) among the clusters using edgeR [30, 31]. This program applies a Generalized Linear Model (GLM) to accommodate an experimental design where the count data are modeled with a negative binomial (NB) distribution [32]. The components were filtered if they expressed a minimum of one count per million (CPM) mapped reads (~8–19 mapped reads per contig) in all seven samples that were sequenced with the mixed tissues. Genes with an FDR < 0.05 based on the Benjamin-Hochberg multiple test correction were identified as being differentially expressed. Only the DEGs in all the pairwise comparisons were included in the following analysis. Based on expression level of these mutual DEGs, we performed a partitioning clustering analysis using a gap statistic approach to estimate the number of clusters (K) as implemented in the clusGap function in R package cluster v1.15.2 [33]. Expression levels of genes assigned to clusters were plotted using *plot_expression_patterns.pl* script provided by the Trinity pipeline [34, 35].

### KEGG pathways and GO enrichment analysis

All the gene functional enrichment analyses of FPGs, DEGs and HEGs were performed with the DAVID Functional Annotation tool (DAVID 6.8 Oct. 2016) [36, 37]. We focused on genes that were annotated in the Kyoto Encylopedia of Genes and Genomes (KEGG) pathways and Gene Ontology (GO) biological process categories. To deeply investigate the functions of genes, all functions enriched in more than two genes would be retained and examined regarding whether it was associated with high-altitude adaptation. Moreover, only the functions enriched in at least two types of genes (FPGs, DEGs and HEGs) would be considered as necessary for environmental adaptation in *C. macropterus*.

## Results

### Sequencing output and assembly

The number of reads per mixed sample after adaptor removal and quality trimming ranged from 40,984,398 to 51,811,346 (Additional file 1: Table S1). After filtering out the redundancy, the de novo assembled transcriptome contained 59,240 unigenes. To assess the quality of the reference transcriptome, all 13 mitochondrial protein-coding genes available for *C. macropterus* in the NCBI database were downloaded and mapped to our reference transcriptome using BLASTN with an E value cutoff of 1 × 10^−10^. Of all the protein-coding genes present in our assembled transcriptome, only four bases were lost at the end of Cytochrome c oxidase II (COX2). In the annotation, 35,408 unigenes exhibited significant BLAST results against the nr database. In total, 10,190 contigs were successfully annotated with GO terms using BLAST2GO.

### Accelerated evolution of *C. macropterus* and adaptive genes detection

*C. macropterus*, *P. fulvidraco* and *G. maculatum* have 1304, 1023 and 948 orthologues with the core-orthologues database, respectively. In total, there are 747 mutual orthologous genes. After alignment and trimming for quality control, 598 orthologous genes were kept for the subsequent evolutionary analysis.

**Fig. 2:**
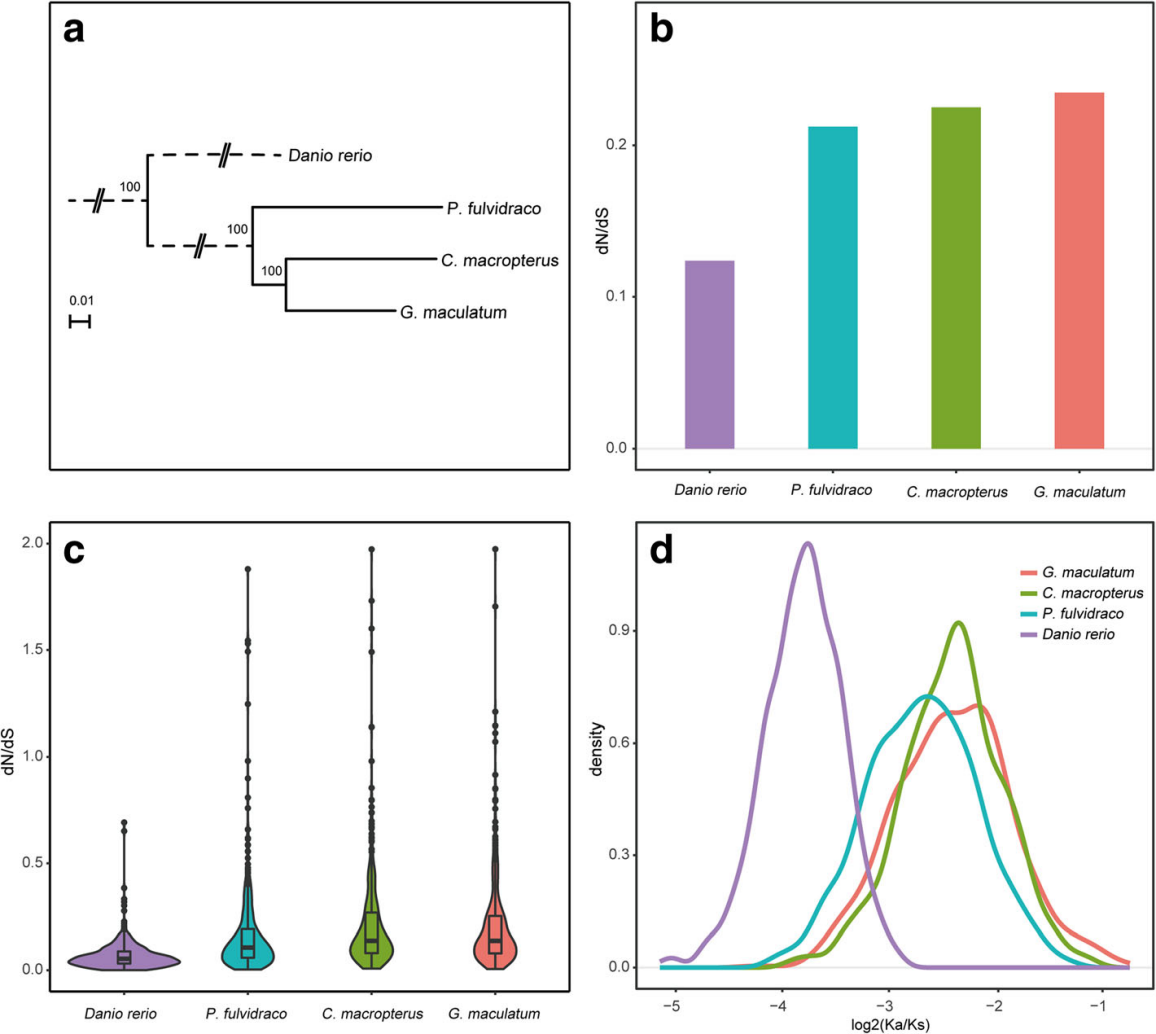
Phylogenetic analysis and evolutionary rate estimation. **a** Phylogenetic tree used in this study. **b** Ka/Ks ratio based on concatenated alignments of all orthologues. **c** The average Ka/Ks ratio based on each orthologue. **d** The average Ka/Ks ratio based on 1000 concatenated alignments constructed from ten randomly chosen orthologues

Based on the constructed phylogenetic tree (Fig. 2a), we evaluated the ratio of nonsynonymous to synonymous substitutions (Ka/Ks) in each orthologue to compare the different selection constraints among the branch of *C. macropterus* and the other two catfish species. Using the free ratio model, we detected that the Ka/Ks value of *C. macropterus* was significantly higher than that of *P. fulvidraco* (Wilcoxon rank sum test, *P* = 4.29e-06) but lower than that of *G. maculatum* (Wilcoxon rank sum test, *P* = 0.987) (Fig. 2b and c). In addition, 1000 concatenated alignments were constructed from ten randomly chosen orthologues, which also indicated the same results (*C. macropterus* vs. *P. fulvidraco*, *P* < 2.2e-16; *C. macropterus* vs. *G. maculatum*, *P* = 0.8721) (Fig. 2d).

To identify the lineage-specific accelerated GO categories of the *C. macropterus* branch, we calculated the mean Ka/Ks ratios and compared these values to those obtained for *P. fulvidraco* and *G. maculatum* to determine whether higher altitudes accelerated evolution. Consequently, we obtained 108 GO terms in which more than five orthologues were involved. The number of GO categories with a higher mean Ka/Ks value increased with increasing altitude (Additional file 2: Figure S1). The “mitochondrial matrix” and “metabolic process” categories in *G. maculatum* were significantly higher than in *P. fulvidraco*, which had not been found in the comparison of *C. macropterus* and *P. fulvidraco*.

In the set of 598 orthologous genes, we identified 450 FEGs and 233 PSGs from the *C. macropterus* branches after correcting for multiple testing. One hundred ninety-eight FPGs (genes overlapped in FEGs and PSGs) (Additional file 1: Table S2) were used in the subsequent analysis.

### Clustering analyses of samples

**Fig. 3:**
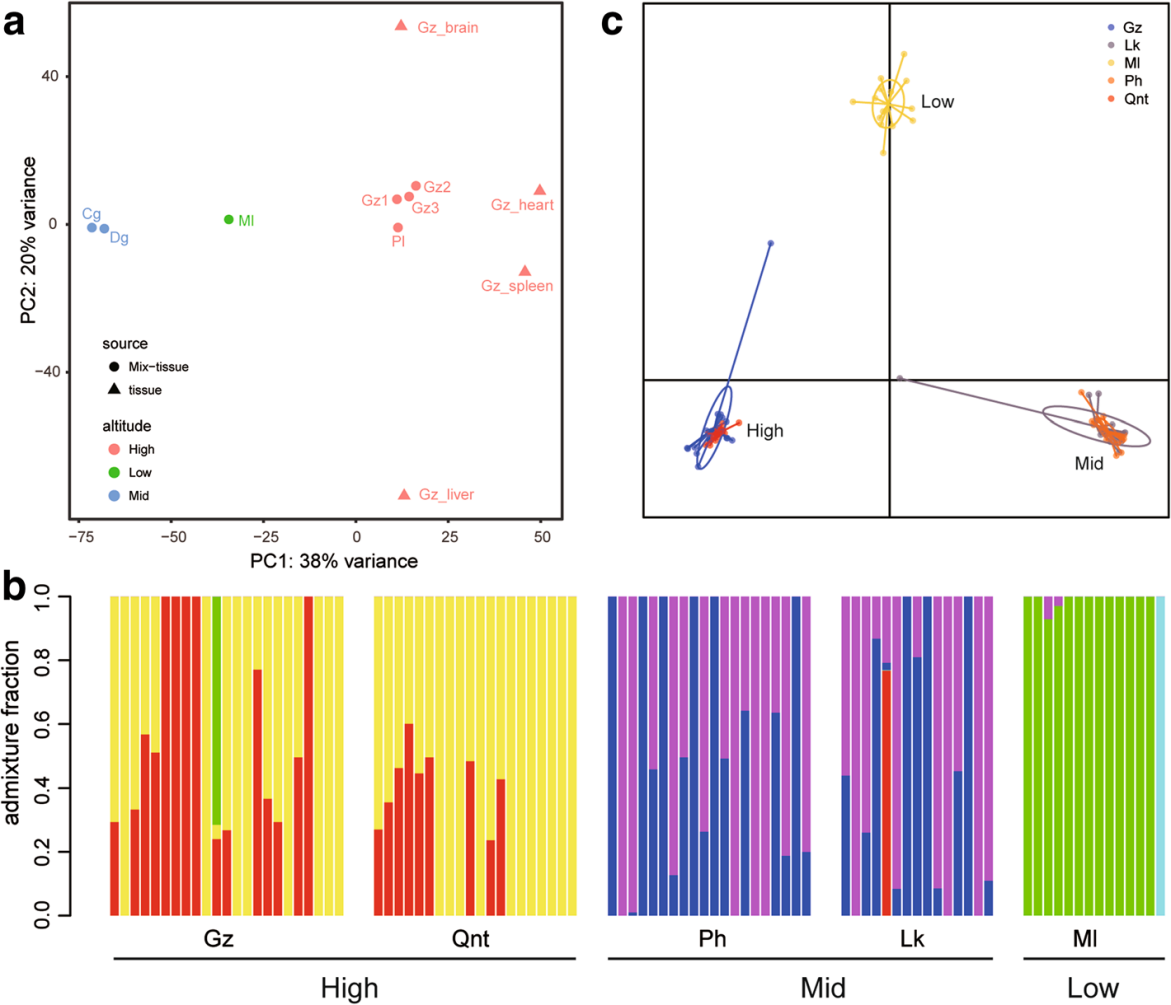
Sample clustering. **a** PCA plots show the clustering of samples based on the variations in the gene expression. **b** Genetic clustering graph of the number of clusters (K = 6). Each colour represents a different genetic cluster. Bar graphs represent the average probability of membership (y-axis) for each individual. **c** DAPC plot based on the SNP dataset of eight populations along the Nujiang River

Based on the gene expression, PCA indicated that 38% of the variance could be explained by principal component 1 (PC1) and 20% could be explained by PC2. The results showed that these individuals could be divided into three clusters. Gz1–3 and Pl from upstream tributaries belonged to cluster 1 (High), Cg and Dg from the main stem belonged to cluster 2 (Mid), and Ml from the downstream tributary belonged to cluster 3 (Low) (Fig. 3a).

After mapping RAD tags to the assembled transcriptome and merging all the loci from each individual, we obtained 28,768 consensus loci, and 155 tags with at least one SNP (the SNP loci must present in all six populations) were included in the subsequent population analyses. The genetic structure revealed that K = 6 had the lowest cross-validation (Additional file 2: Figure S2) of the five populations. Almost all individuals from the upper tributaries (Qnt and Gz) had two genetic structures (red and orange). Most individuals from the mid stem (Lk and Ph) had two other genetic structures (purple and blue), and most individuals from the downstream tributary (Ml) had only one genetic structure (green) (Fig. 3b). In fact, all individuals could be grouped into three clusters, because the components of the genetic structures in Qnt and Gz (or Lk and Ph) were uniform. This arrangement was also indicated by the DAPC plot (Fig. 3c), which shows that the five populations formed three groups based on the localities.

### Examination of differentially expressed genes

**Fig. 4:**
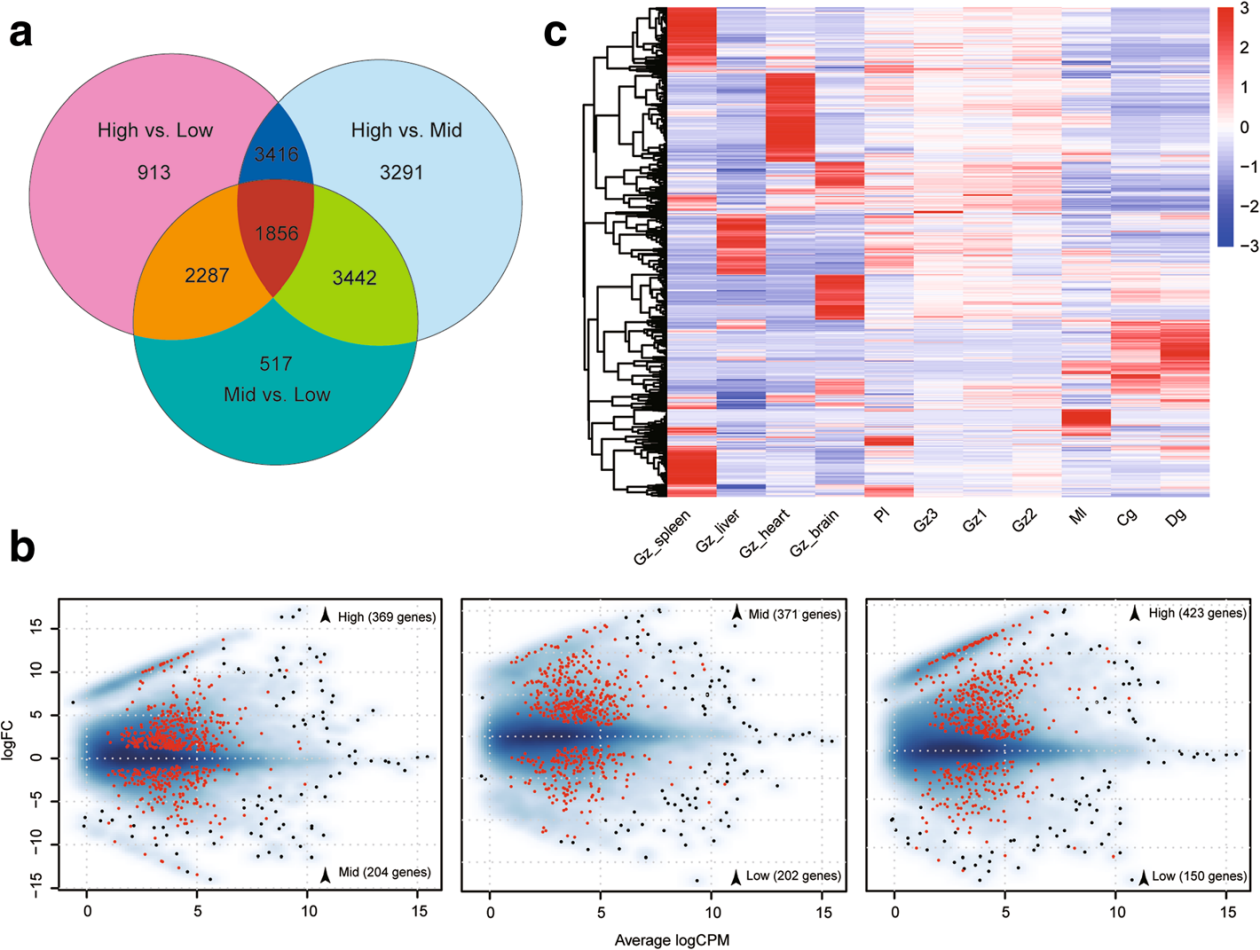
Examination of the DEGs and HEGs. **a** Venn diagram of the differentially expressed contigs at high, mid and low altitudes. **b** Smear plots showing 573 mutual DEGs in red in each pairwise comparison. A smooth scatter was used to convert the number of points in each plot coordinate into a vector of colours representing the local point density. Darker shades of blue represent higher density of points. Only below a specific density or in case of significance the dot is drawn. **c** Heat map of the top 500 most highly expressed genes in each sample. Colours represent the FPKM value of the gene expression after scaling and centring

By comparing the patterns of differential gene expression among three clusters (High, Mid and Low), a total of 15,722 contigs were inferred as differentially expressed genes (FDR < 0.05) in at least one pairwise comparison (Additional file 2: Figure S3). Of these, 1856 contigs were differential expressed among all comparisons, of which 573 were in the list of reciprocal best BLAST hits with zebrafish (Fig. 4a, Additional file 1: Table S3). The number of DEGs that highly expressed in individuals from high altitudes were always more than mid or low altitude individuals (Fig. 4b, Additional file 2: Figure S3). Among the mutual 573 DEGs, we found 369 and 423 genes were highly expressed in High when compared to Mid and Low (Fig. 4b), and the number of highly expressed genes were more in Mid than that in Low (371 vs. 202). We then defined co-expressed sets of genes using partitioning clustering analysis in the 573 mutual DEGs. For this analysis, the PAM (Partitioning Around Medoids) clustering algorithm assumed the optimal value of k = 3 using 1000 bootstrap replicates resampling (Additional file 2: Figure S4). The results indicated three major expression patterns in the 573 mutual DEGs (Additional file 2: Figure S5). Of the three patterns, a pattern showed genes were more expressed in high altitudes (Pl, Gz1, Gz2 and Gz3) than that in low altitudes (Mid (Cg and Dg) and Low (Ml)) (Additional file 2: Figure S5), which contained maximum number of genes (155 genes, Additional file 1: Table S4) in the three major expression patterns. The above 573 mutual DEGs would be applied into the next gene functional analysis.

### Detection of highly expressed genes in high altitude

There were 1377 HEGs after selecting and merging the top 500 highly expressed genes (HEGs) in each sequencing library. In the expression profiles of these genes (Fig. 4c), we observed that certain genes appeared to be strikingly highly expressed in the tissues (spleen, liver, heart and brain of Gz), and their expressions were progressively decreasing along altitude decreasing (gene were higher expressed in highland (Pl, Gz1, Gz2 and Gz3) than that in lowland (Ml, Cg and Dg)). To obtain this type of genes as much as possible, we selected the top 400 HEGs in each tissue, which left 657 unigenes after removing redundancy. Of these, 220 were the best reciprocal BLAST hits with zebrafish (Additional file 1: Table S5), and these 220 HEGs would be used in the following gene functional enrichment analysis to detect the underlying genomic signals of high-altitude adaptation.

### Gene function enrichment analysis

In the KEGG pathways enrichment analysis, we focused on representative key pathways (top 10 enriched gene number) in FPGs, DEGs and HEGs (Additional file 2: Figure S4). In the three gene datasets, HEGs and DEGs were significantly enriched in 9 and 2 pathways, and FPGs were not significantly enriched in any pathways. The results indicated the highest number of genes annotated to the metabolic pathways, and approximately 15% of the HEGs were significantly involved in oxidative phosphorylation (*P* = 0.027). It was interesting to find that six HEGs (e.g, COX8B, COX1 and UGCRG) were involved in cardiac muscle contraction (*P* = 0.085). In addition, we detected genes related to the biosynthesis of antibiotics in all gene datasets.

**Fig. 5:**
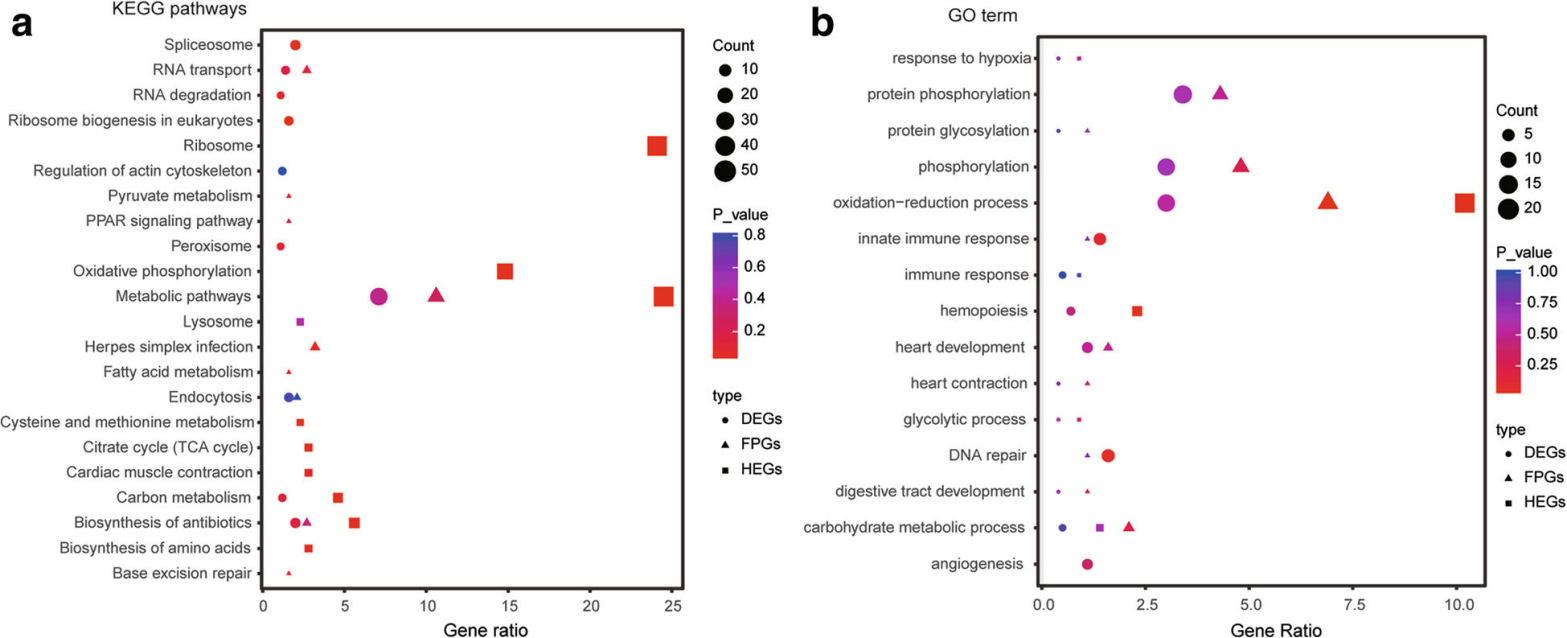
Enrichment function analysis of the FPGs, DEGs and HEGs. **a** The top ten KEGG pathways (sorted by enriched gene number) in each dataset. **b** Fifteen GO terms that are involved in high-altitude adaptation. The gene ratio is representative of the gene numbers in each dataset

To capture genes that might participate in high-altitude adaptation, we also performed a GO enrichment analysis to link the FPGs, DEGs and HEGs to underlying biological processes. The results showed that the three gene datasets were associated with 254 biological processes (enriched at least two genes), and 15 GO terms (emerged at least two gene datasets) might be associated with high altitude adaptation (Fig. 5b, Table 2 and Additional file 1: Table S6). In these genes associated with the 15 GO terms, a large number of genes were involved in the “oxidation-reduction”, “phosphorylation process” and “protein phosphorylation”. The other functions, in contrast, were enriched in far fewer genes. For example, two HEGs (MB and PGK1) and two DEGs (HSPA5 and PGK1) were annotated to “response to hypoxia”, while nine DEGs and two FPGs were (TGD.2 and APEX2) enriched for “DNA repair”.

**Table 2:** GO terms associated with high altitude adaptation in *C. macropterus*

GO terms	Gene lists number	DEGs	FPGs	HEGs
Oxidation-reduction process	3	17	**13**	**22**
Carbohydrate metabolic process	3	3	4	3
Immune response	2	3	–	2
Glycolytic process	2	2	–	2
DNA repair	2	9	2	–
Protein glycosylation	2	2	2	–
Phosphorylation	2	17	9	–
Angiogenesis	2	6	2	–
Response to hypoxia	2	2	–	2
Hemopoiesis	2	4	–	**5**
Digestive tract development	2	2	2	–
Protein phosphorylation	2	19	8	–
Innate immune response	2	8	2	–
Heart contraction	2	6	3	–
Heart development	2	3	2	–

The table includes the number of datasets related to this GO term, and the number of genes associated with this GO term in FPGs, DEGs and HEGs. The numbers in bold represent the significantly enriched genes (*P* < 0.05)

## Discussion

In the present study, we sequenced and assembled a comparable intact transcriptome of *C. macropterus* using eleven RNA-seq libraries. We calculated the evolutionary rates of *P. fulvidraco*, *C. macropterus* and *G. maculatum* and then identified the fast-evolving genes and positively selected genes of *C. macropterus*. By estimating the gene expression, we also acquired highly expressed genes from the tissues of upstream tributaries, and calculated differentially expressed genes among three *C. macropterus* clusters. Through function enrichment analysis of these genes, we identified the underlying genetic signals that might be responsible for high-altitude adaptation in *C. macropterus*.

### Accelerated evolutionary rates along increased altitude

Through estimating the ratio of nonsynonymous to synonymous substitutions, we found that the *C. macropterus* lineage exhibited an accelerated evolutionary rate compared with that of *P. fulvidravo* in the lowland areas (< 100 m). However, *G. maculatum* in the higher altitude (3800–4000 m) showed accelerated evolution rates relative to *C. macropterus*. In addition, the number of GO terms with a higher evolutionary rate were increased as altitude increase. Therefore, *C. macropterus* did display genome-wide accelerated evolution. The evolutionary rate might speed up with increased altitude to cope with the hasher environment at the higher altitude.

### Genetic difference of *C. macropterus* along the Nujiang River basin

According to the results of the sample clustering, *C. macropterus* along the Nujiang River basin could be divided into 3 clusters (High, Mid and Low). The dN/dS ratio was originally developed for application to distantly diverged sequences [38–40]. Therefore, the direct comparison of the evolution rates of three clusters in a phylogenetic tree might result in errors. To investigate the genetic difference in the three clusters, we calculated the expression levels of genes to obtain pairwise DEGs.

The GO enrichment analysis indicated that DEGs were significantly enriched in 12 functional categories. A previous study showed that positively selected genes of *Rana chensinensis* inhabiting higher elevations were significantly enriched in “peroxisome organization” compared to the *R. kukunoris* inhabit lower elevation [41]. The DEGs of our study were also associated with “peroxisome organization” (3 genes, *P* = 0.0039). And there were three significant terms (“central nervous system development” (7 genes, *P* = 0.0085), “cellular response to DNA damage stimulus” (9 genes, *P* = 0.0086) and “DNA damage checkpoint” (3 genes, *P* = 0.05)), which were involved in high altitude adaptation [42, 43]. In the largest gene expression pattern (cluster 1, Additional file 2: Figure S5), we have detected 0, 1, 4 and 4 genes associated with “peroxisome organization”, “DNA damage checkpoint”, “central nervous system development” and “celluar response to DNA damage stimulus”, respectively. It seemed that altitude increases might result in the differentiation of *C. macropterus*. With the rise of altitude, oxidation reactions and stress responses are more and more important to regulate factors in animals, which might need more expression of relevant genes.

### Energy metabolism of *C. macropterus*

High energy metabolism is necessary to survive in high-altitude habitats [44–46]. Through enrichment analysis of the FPGs, HEGs and DEGs, the present study identified many genes that were directly associated with energy metabolism processes (e.g., “carbohydrate metabolic process”, “glycolytic process” and “protein glycosylation”). In addition, we identified some genes related to blood circulation and heart functions (e.g., “angiogenesis”, “hemopoiesis” and “heart development”).

Of these genes, GAPDHS (glyceraldehyde-3-phosphate dehydrogenase, spermatogenic) and PGK1 (phosphoglycerate kinase 1) were highly expressed in high-altitude tissues and differentially expressed among the three clusters, which were involved in “glycolytic process”. Evidence shows that GAPDHS encodes a protein belonging to the glyceraldehyde-3-phosphate dehydrogenase (GAPDH) family of enzymes, and GAPDHS is post-transcriptionally regulated and is up-regulation in hypoxia endothelial cells [47, 48]. For the PGK1 gene, a previous study reported that it is widely expressed in response to hypoxia [49]. Excluding PGK1, we also found 2 other genes (MB and HSPA5) that were annotated in “response to hypoxia” [50, 51] in HEGs and DEGs, respectively. The related reports indicated a significantly higher MB (myoglobin) protein content after training in hypoxia [52], and hypoxia decreases the expression of heat shock proteins 5 (HSPA5) in human microvascular endothelial cells [53].

### Immune response and DNA repair of *C. macropterus*

The responses to hypoxia and UV radiation are crucial for organisms to adapt to high-altitude environments. In *C. macropterus*, we found 2 HEGs (C9 and BLNK) and 3 DEGs (TNFAIP3, NRROS and BLNK) that were involved in the “immune response”, as well as 2 FPGs (NFKB2 and PTK2BA) and 8 DEGs (e.g. NRROS, FER and ANKHD1) associated with the “innate immune response”. BLNK (B-cell linker) and NFKB2 (nuclear factor of kappa light polypeptide gene enhancer in B-cells 2) are two genes related to B cells, and B cells function in the humoral immunity component of the adaptive immune system by secreting antibodies, which would help *C. macropterus* in the adaptation to high-altitude environments. For BLNK^−/−^ mice, the mutant B cells failed to mature and are non-responsive to B cell receptor cross-linking in terms of proliferation [54]. Hypoxia-inducible factor (HIF)-1α suppression in hypoxic conditions was associated with the down regulation of transcription factor genes (e.g. NFKB2) [55]. Reactive oxygen species (ROS) produced by phagocytes are essential for host defence against bacterial and fungal infection. NRROS (negative regulator of reactive oxygen species), named the negative regulator of reactive oxygen species, limits ROS generation by phagocytes during inflammatory responses.

DNA damage induced by hypoxia and UV radiation might be universal at high-altitudes [56, 57], species in high altitude need relevant functions to repair the DNA damages. There were 2 FPGs (APEX2 and TGD.2) and 9 DEGs (e.g., RNF8, MTOR and TONSL) that were related to “DNA repair”. We identified that 4 DEGs (i.e., WRN, RFC1, EMSY and APTX) were highly expressed in individuals from high-altitude compared to that of Mid and Low (Additional file 1: Table S4, the cluster 1 in Additional file 2: Figure S5). For example, APTX (aprataxin) is a 342 amino acid protein that localizes to the nucleus and nucleolus [58, 59], which is associated with both the DNA single-strand and double-strand break repair machinery. Detailed examination of three distinct domains in APTX elucidated the repair role played by APTX and its relevance for maintaining genome integrity in neuronal tissues [60]. Along the altitude increase, DNA repair in *C. macropterus* might become stronger in the harsher environment.

### Controversial issue and limitations

The genetic structure revealed that K = 6 had the lowest cross-validation (Additional file 2: Figure S2) in five *C. macropterus* populations along the Nujiang River Basin, which is different from the results obtained in our previous study (K = 3) [25]. Considering that most transcripts might be not sequenced because of the absence of a special restriction site, we could only obtain 155 tags with SNPs, which was much fewer than the 1679 tags in our previous study, which might have resulted in a reduced resolution. The difference might also have resulted from some type of complicated interaction due to the biases associated with transcriptome assembly and RAD-seq. In addition, the DEGs results might be questionable because of the small sample size in our analysis. We only sampled one individual from low-altitude and two individuals from mid-altitude, which might result in occasional errors. The results would be much more reliable if we sampled more individuals, especially individuals in the mid and low clusters. And measuring more environmental factors (e.g. oxygen concentration, temperature and components of water) might be helpful for elucidating the expression patterns of genes in these locations.

## Conclusions

In this study, we reported that the transcriptome of *C. macropterus*, investigated the genetic mechanism of high-altitude adaptation in *C. macropterus*, and detected differentially expressed genes within three clusters. By comparing the evolutionary rates, we found that species from higher altitudes might evolve faster than species from the lowlands. Based on functional enrichment analysis of the FPGs, HEGs and DEGs, the results indicated that many genes were involved in energy metabolism and hypoxia, which might be an important resource to study adaptations to high-altitude environment.

## Additional files

Additional file 1: Table S1. Sequencing output result of all eleven mixed samples. Q20 represents the percentage of quality score of bases that above 20. Q30 represents the percentage of quality score of bases that above 30. **Table S2.** FPGs (The overlaps of genes of the fast evolving genes (FEGs) and positively selected genes (PSGs)). **Table S3.** Mutual DEGs (differentially expressed genes) that best reciprocal BLAST hits with zabrafish in all pairwise comparisons (High vs. Mid, Mid vs. Low and High vs. Low). **Table S4.** Genes in cluster 1 (the largest gene group in the resluts of co-expression analysis) which were highly expressed in individuals from high-altitude. **Table S5.** Highly expressed gene in tissues from high-altitude. **Table S6.** GO terms enriched by genes of FPGs, DEGs and HEGs. (XLSX 73 kb)

Additional file 2: Figure S1. Scatter plot of the mean Ka/Ks ratio with more than five orthologues for each GO category in *C. macropterus*, *P. fulvidraco*, *G. maculatum,* and *D. rerio*. The slope of oblique line is 45°, points above this line presented GO terms with higher Ka/Ks ratios in species of y axis. If not, points presented GO terms with higher Ka/Ks ratios in species of x axis. Red points with annotated text represent GO categories with significantly higher Ka/Ks ratios. Black arrows symbolize the number of genes with higher Ka/Ks ratios. **Figure S2.** ADMIXTURE cross-validation approach to choosing the K parameter – the postulated number of ancestral populations. The error estimates are based on the 10-fold cross-validation. **Figure S3.** Smear plots showing the number of differentially expressed contigs (FDR < 0.05) in red in each pairwise comparison. A smooth scatter was used to convert the number of points in each plot coordinate into a vector of colours representing the local point density. Darker shades of blue represent higher density of points. Only below a specific density or in case of significance the dot is drawn. **Figure S4.** Gap statistic for the expression of 573 mutual DEGs using 1000 bootstrap replicates resampling and the PAM clustering alogorithm. **Figure S5.** Three major clusters of genes with similar expression pattern. Expression of genes in cluster 1 were higher in samples from high altitudes than that from mid and low altitudes. (DOCX 1132 kb)
