# Supplementary material for: Evidence of high-altitude adaptation in the glyptosternoid fish, Creteuchiloglanis macropterus from the Nujiang River obtained through transcriptome analysis
Source: BMC Evol Biol. 2017 Nov 23;17:229. doi: 10.1186/s12862-017-1074-0 (PMC5701497; doi:10.1186/s12862-017-1074-0)

Fig. S1. Scatter plot of the mean Ka/Ks ratio with more than five orthologues for each GO category in *C. macropterus*, *P. fulvidraco*, *G. maculatum,* and *D. rerio*. The slope of oblique line is 45°, points above this line presented GO terms with higher Ka/Ks ratios in species of y axis. If not, points presented GO terms with higher Ka/Ks ratios in species of x axis. Red points with annotated text represent GO categories with significantly higher Ka/Ks ratios. Black arrows symbolize the number of genes with higher Ka/Ks ratios.


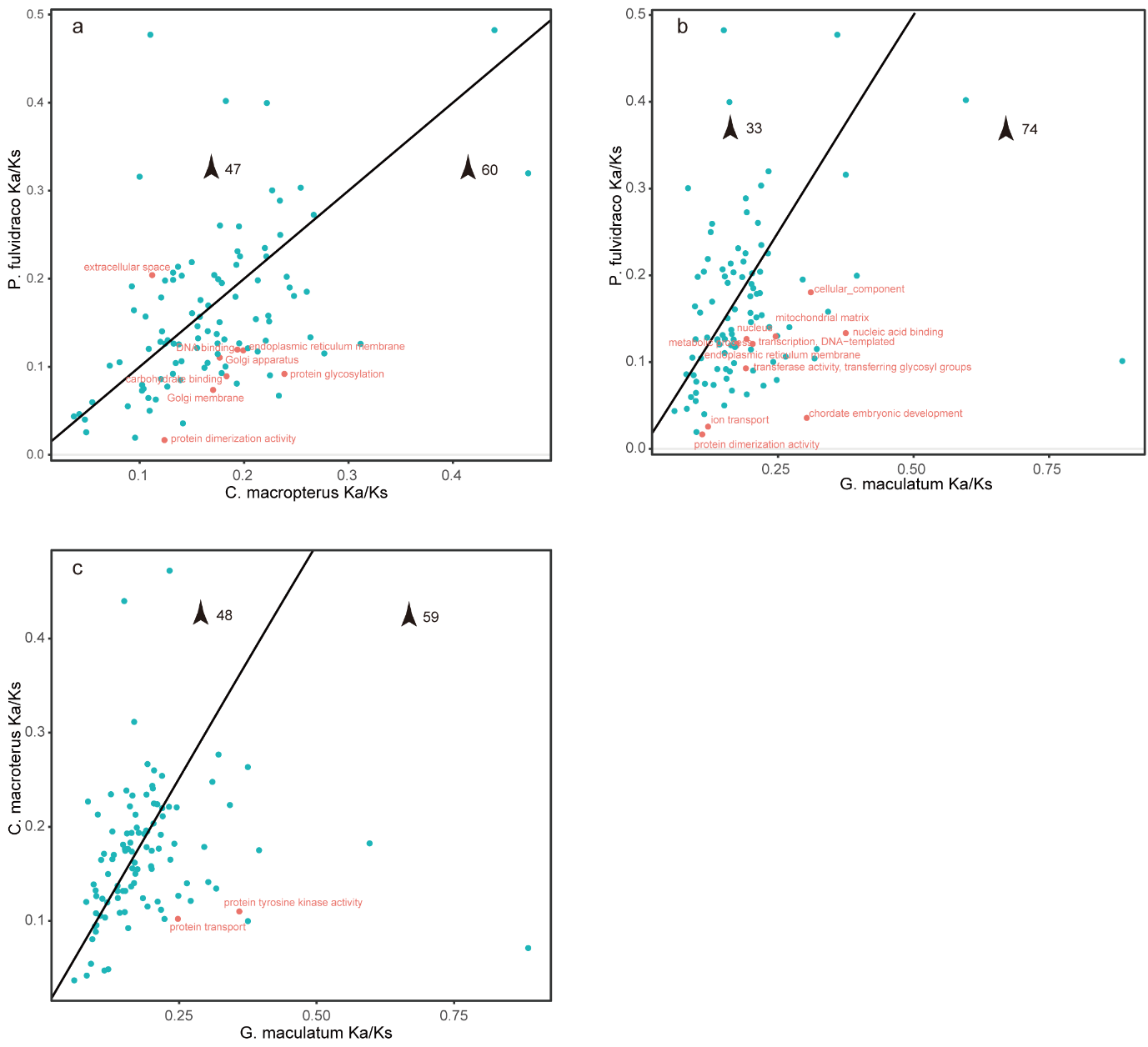


Fig. S2. ADMIXTURE cross-validation approach to choosing the K parameter – the postulated number of ancestral populations. The error estimates are based on the 10-fold cross-validation.


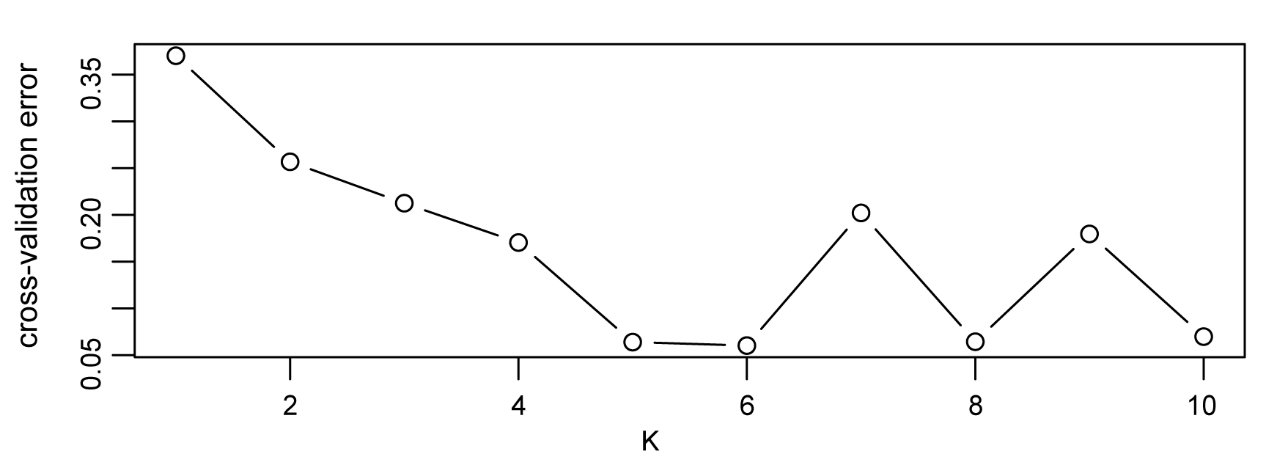


Fig. S3. Smear plots showing the number of differentially expressed contigs (FDR<0.05) in red in each pairwise comparison. A smooth scatter was used to convert the number of points in each plot coordinate into a vector of colours representing the local point density. Darker shades of blue represent higher density of points. Only below a specific density or in case of significance the dot is drawn.


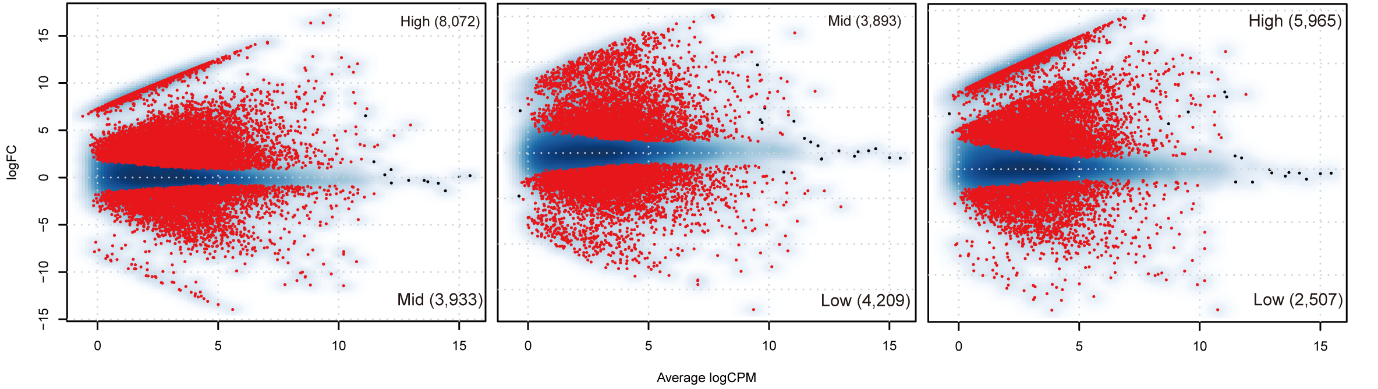


Fig. S4 Gap statistic for the expression of 573 mutual DEGs using 1,000 bootstrap replicates resampling and the PAM clustering alogorithm.


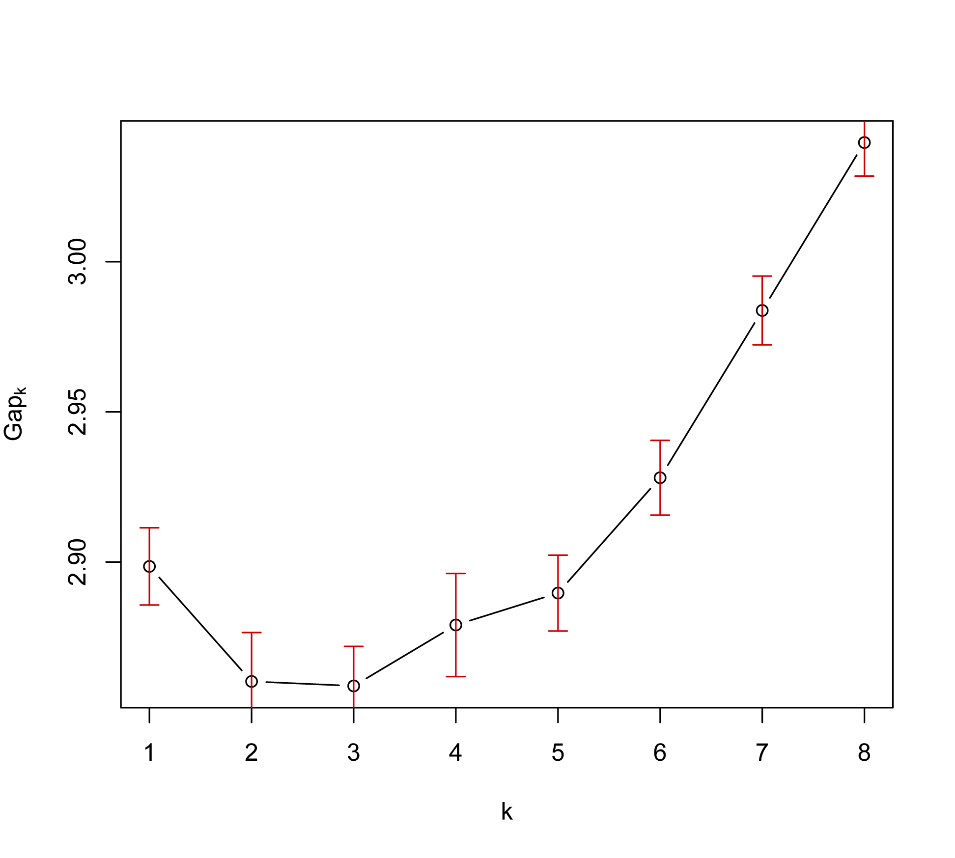


Fig. S5 Three major clusters of genes with similar expression pattern. Expression of genes in cluster 1 were higher in samples from high altitudes than that from mid and low altitudes.


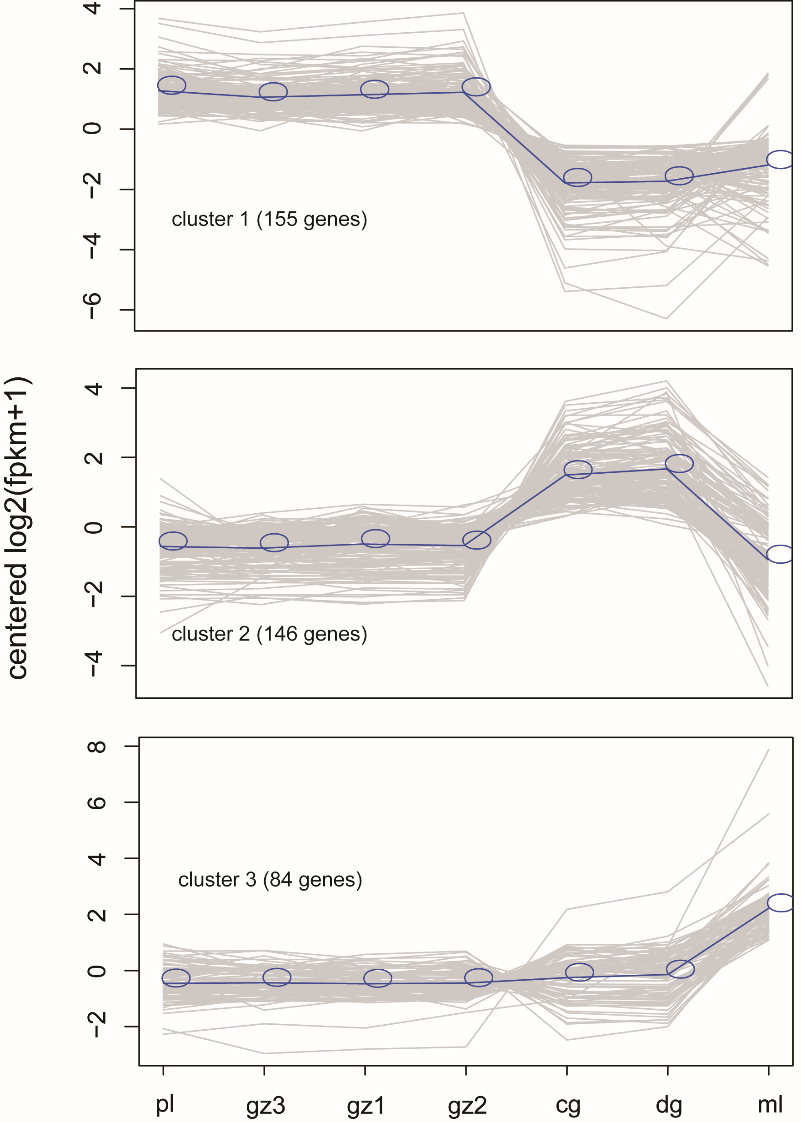

Supplement: Supplementary file 2 — Scatter plot of the mean Ka/Ks ratio with more than five orthologues for each GO category in C. macropterus, P. fulvidraco, G. maculatum, and D. rerio. The slope of oblique line is 45°, points above this line presented GO terms with higher Ka/Ks ratios in species of y axis. If not, points presented GO terms with higher Ka/Ks ratios in species of x axis. Red points with annotated text represent GO categories with significantly higher Ka/Ks ratios. Black arrows symbolize the number of genes with higher Ka/Ks ratios. Figure S2. ADMIXTURE cross-validation approach to choosing the K parameter – the postulated number of ancestral populations. The error estimates are based on the 10-fold cross-validation. Figure S3. Smear plots showing the number of differentially expressed contigs (FDR < 0.05) in red in each pairwise comparison. A smooth scatter was used to convert the number of points in each plot coordinate into a vector of colours representing the local point density. Darker shades of blue represent higher density of points. Only below a specific density or in case of significance the dot is drawn. Figure S4. Gap statistic for the expression of 573 mutual DEGs using 1000 bootstrap replicates resampling and the PAM clustering alogorithm. Figure S5. Three major clusters of genes with similar expression pattern. Expression of genes in cluster 1 were higher in samples from high altitudes than that from mid and low altitudes. (DOCX 1132 kb) [file 12862_2017_1074_MOESM2_ESM.docx]
